# Signatures of Diversifying Selection in European Pig Breeds

**DOI:** 10.1371/journal.pgen.1003453

**Published:** 2013-04-25

**Authors:** Samantha Wilkinson, Zen H. Lu, Hendrik-Jan Megens, Alan L. Archibald, Chris Haley, Ian J. Jackson, Martien A. M. Groenen, Richard P. M. A. Crooijmans, Rob Ogden, Pamela Wiener

**Affiliations:** 1 The Roslin Institute and Royal (Dick) School of Veterinary Studies, University of Edinburgh, Edinburgh, United Kingdom; 2 Animal Breeding and Genomics Centre, Wageningen UR, Wageningen, The Netherlands; 3 MRC Human Genetics Unit, MRC Institute of Genetics and Molecular Medicine, University of Edinburgh, Edinburgh, United Kingdom; 4 Wildgenes Laboratory, Royal Zoological Society of Scotland, Edinburgh, United Kingdom; The University of Queensland, Australia

## Abstract

Following domestication, livestock breeds have experienced intense selection pressures for the development of desirable traits. This has resulted in a large diversity of breeds that display variation in many phenotypic traits, such as coat colour, muscle composition, early maturity, growth rate, body size, reproduction, and behaviour. To better understand the relationship between genomic composition and phenotypic diversity arising from breed development, the genomes of 13 traditional and commercial European pig breeds were scanned for signatures of diversifying selection using the Porcine60K SNP chip, applying a between-population (differentiation) approach. Signatures of diversifying selection between breeds were found in genomic regions associated with traits related to breed standard criteria, such as coat colour and ear morphology. Amino acid differences in the *EDNRB* gene appear to be associated with one of these signatures, and variation in the *KITLG* gene may be associated with another. Other selection signals were found in genomic regions including QTLs and genes associated with production traits such as reproduction, growth, and fat deposition. Some selection signatures were associated with regions showing evidence of introgression from Asian breeds. When the European breeds were compared with wild boar, genomic regions with high levels of differentiation harboured genes related to bone formation, growth, and fat deposition.

## Author Summary

The domestic pig, an important source of protein worldwide, was domesticated from the ancestral wild boar in multiple locations throughout the world. In Europe, local types were developed following domestication, but phenotypically distinct breeds only arose in the eighteenth century with the advent of systematic breeding. Recently developed molecular tools for pigs (as well as other livestock species) now allow a genetic characterisation of breed histories, including identification of regions of the genome that have been under selection in the establishment of breeds. We have applied these tools to identify genomic regions associated with breed development in a set of commercial and traditional pig breeds. We found strong evidence of genetic differentiation between breeds near genes associated with traits that are used to define breed standards, such as ear morphology and coat colour, as well as in regions of the genome that are associated with pork production traits. It is well documented that crosses with Asian pigs have been used to modify European breeds. We have found evidence of genetic influence from Asian pigs in European breeds, again in regions of the genome associated with breed standard characteristics, including ear shape and coat colour, as well as production traits.

## Introduction

The domestic pig is an important livestock species and an important protein source worldwide. The pig originated from the wild boar, *Sus scrofa*, by multiple independent domestications, mainly in Asia Minor, Europe and East Asia [1], [2]. Domestication and subsequent selective pressures altered the behaviour and phenotypic characteristics of these animals [3]. Local pig types were developed in Europe and Asia after domestication, but the development of phenotypically distinct breeds chiefly occurred with the commencement of organised breeding in the 18^th^ century [4].

Strict organised breeding was adopted to improve and develop livestock breeds and Britain in particular was a main centre of the early improvement of pig breeds [5], [6], as a reaction to increasing demand for meat in the wake of the industrial revolution. From the 18^th^ century pig breeds were selectively bred for specific production traits such as early maturation, rapid growth and increased prolificacy. In addition, the coat colour phenotype (which includes both skin and hair pigmentation) was another morphological trait often used during the selective breeding process. Substantial morphological changes occurred in breeds over a short period of time, resulting in the development of numerous distinct pig breed phenotypes in Britain. Charles Darwin commented on the rapid morphological changes in pig breeds at that time: “Chiefly, in consequence of so much crossing, some well-known breeds have undergone rapid changes; thus, according to Nathusius […] the Berkshire breed of 1780 is quite different from that of 1810; and, since this latter period, at least two distinct forms have been borne the same name.” [4]. Although breeds tended to be formed by complex crossing with numerous other breeds, including a number from Asia, to introduce desirable traits [4]–[6], after improvement the breeds were kept distinct, resulting in highly specialised phenotypically distinct and genetically differentiated pig breeds [7]. From the 20^th^ century, with the recognition of the benefits of genetic improvement and changing consumer preferences, certain pig breeds experienced further strong selection for lean meat content, muscularity and enhanced reproduction [5], [6].

To better understand the genetic basis for phenotypic variation in the pig, studies have focused on important traits relevant to the breed development process with the aim of identifying, characterising and mapping candidate genes, and subsequently identifying the underlying causal mutations and allelic differences between breeds [8], [9]. Studies mapping quantitative trait loci (QTL) have particularly focused on muscle growth. Fine mapping of one of these regions (SSC2) identified a causal mutation in the *IGF2* gene, where a single nucleotide change is associated with high muscle content in some commercial pig populations [10]. The level of fat on the carcass is also a production trait of economic impact and QTL studies have mapped loci associated with fat deposition to various chromosomes, in particular SSC4 and SSC7 [11], [12]. Reproductive traits have received attention in pigs with several genes investigated in relation to litter size and the number of teats (*ESR*, *PTHLH* and *PTHR1*) [13]. Coat colour is considerably varied amongst breeds within domesticated animal species and investigations into the genetics of pigmentation have identified numerous loci influencing these traits [8], [9]. Variation at two genes, *KIT* and *MC1R*, is associated with a variety of pig breed colour types including red, black and white colouring and belted and spotted phenotypes [14]–[16].

With growing genomic resources, selection mapping approaches are increasingly being implemented to identify genetic variants that underlie the phenotypic diversity in domesticated animals. These approaches involve scanning the genome for levels of population differentiation and diversity [17]. Genome-wide scans for signatures of diversifying selection in livestock species have detected signals revealing candidate genes related to morphological variation such as body size, skeletal formation, cranial structure and coat patterns, and production traits such as muscle conformation and milk yield [18]–[25].

To further explore the genetic variation underlying the phenotypic diversity of pig breeds, a genome-wide scan of a diverse set of commercial and traditional British/European pig breeds was performed to identify genomic regions showing signatures of between-breed (diversifying) selection using levels of breed genetic differentiation (F_ST_). Based on these results, sequence data from three candidate regions was analysed to investigate potential causative variants.

## Results

### Signatures of diversifying selection

A genome-wide scan for signatures of selection in 13 European pig breeds (Table 1) was carried out by estimating Wright's F_ST,_ a measure of population genetic differentiation, at each genetic marker. After adopting a sliding window approach, candidate regions that may have experienced diversifying selection were identified by taking the 99^th^ percentile of the empirical distribution of F_ST_–windows (Figure S1). A total of 491 F_ST_–windows per breed were deemed as outlier regions and as many were adjacent SNPs that clustered together, a total of 446 genomic regions displayed strong breed differentiation.

**Table 1:** Samples from pig breeds and wild boar.

	Breed		*N*	Type	Average F_ST_ 4	Sampling^3^
1	Berkshire	BK	29	Traditional	0.139	PigBioDiv and USA
2	British Saddleback	BS	30	Traditional	0.103	PigBioDiv
3	Duroc	DU	26	Commercial	0.163	This study
4	Gloucestershire Old Spots	GLOS	24	Traditional	0.147	PigBioDiv
5	Hampshire	HA	30	Commercial	0.146	PigBioDiv
6	Landrace	LR	27	Commercial	0.126	This study
7	Large Black	LB	30	Traditional	0.127	PigBioDiv
8	Large White	LW	31	Commercial	0.119	This study
9	Mangalica	MA	26	European^1^	0.149	PigBioDiv
10	Middle White	MW	30	Traditional	0.132	PigBioDiv
11	Pietrain	PI	26	Commercial	0.125	This study
12	Tamworth	TA	30	Traditional	0.151	PigBioDiv
13	Welsh	W	33	Traditional	0.115	This study
14	Meishan	ME	24	Asian^2^	0.281	PigBioDiv
15	Wild boar	WB	29	Wild progenitor	0.117	SNP discovery process

1 first imported to Britain from Hungary in 2006,

2 first imported to Britain from China in 1800 s,

3 the sampling protocol is further described in Materials and Methods.

4 Average F_ST_ was calculated as the genome-wide average of the indicated breed against each of the others (for the European breeds), the genome-wide average of Meishan against each of the European breeds (for Meishan), and the genome-wide average of wild boar against each of the European breeds (for wild boar).

### Signatures of selection shared in multiple breeds

The genome-wide scan revealed five genomic regions of extremely high levels of differentiation that overlapped in five or more breeds; all of these regions contain biologically interesting candidate genes (Table 2). One such region was observed in eight breeds on SSC5 (32.32–34.06 Mb). In all but two of the breeds, the peak F_ST_–window (∼32.6–32.8 Mb) overlapped with the genes *WIF1* (32.66–32.72 Mb) and *LEMD3* (32.77–32.89 Mb). This region is orthologous to a region in dogs associated with ear morphology [19], [24]. Another region was detected in five breeds on SSC7 (54.00–57.00 Mb), where at the 97.5^th^ percentile a further four breeds also exhibited a signal. On SSC8, a region of high differentiation spanning 71.84–75 Mb was observed in nine breeds. More striking was the extended region of differentiation on SSC8 spanning 40–75 Mb observed in most breeds, with numerous overlapping and non-overlapping peaks of F_ST_ across a large genomic region on that chromosome (Figure S1), although fewer than five breeds overlapped directly in their peak F_ST_–windows, except in the narrow interval mentioned above. Duroc was the only breed that did not show high levels of differentiation in this region, or even on that chromosome, at either the 99^th^ or 97.5^th^ percentile. Outlier regions were also found on SSC15 (139.60–142.10 Mb), observed in six breeds, and on SSC16 (18.72–20.63 Mb), observed in five breeds.

**Table 2:** Summary statistics for genomic regions with outlier F_ST_-windows that were identified across 5 or more breeds.

*Multiple-breeds*	Chr	Position (Mb)	Total numbers of genes^1^				Candidate Genes	Gene name or proposed function
			Protein-coding	Uncharacterized protein	Non-coding RNA	Pseudogene		
*BK/BS/GLOS/HA/LB/LW/MA/TA*	5	32.32–34.06	7	4	5	1	WIF1	Bone development: inhibitor of the Wnt signalling pathway
							LEMD3	Bone development: operates is BMP signalling
							MSRB3	methionine sulfoxide reductase B3
							HMGA2	high mobility group AT-hook 2 (Body size)
*BS/LR/MA/PI/W*	7	54.00–57.00	14	6	3	1	IL16	Immunity
							TMC3	transmembrane channel-like 3
							ADAMTSL3	Body size, variation
*BK/BS/GLOS*/*HA/LB/MA/PI/TA/W*	8	71.84–75.00	21	7	4	0	SLCA4	solute carrier involved in transport/blood phH
							ADAMTS3	ADAM metallopeptidase with thrombospondin type 1 motif, 3
							AREG	Reproduction
							ANKRD17	ankyrin repeat domain 17 (reproduction)
*BS/LR/LB/LW/MA/TA/W*	15	139.60–142.10	4	2	3	1	LOC100512503	Unidentified
							IRS1	A component of the highly conserved IGF1 signalling cascade pathway that regulates skeletal muscle growth in mammals.
							COL4A4	Bone and cartilage development
*BK/BS/HA/LB/MA/W*	16	18.72–20.63	4	3	2	1	ADAMTS12	ADAM metallopeptidase with thrombospondin type 1 motif, 12
							SLC45A2	Coat colour variation
							PRLR	The prolactin receptor implicated In several reproductive traits including litter size

1 categorization of genes based on Ensembl/Biomart annotation.

### Signals unique to individual breeds

Most extreme genomic regions were observed in fewer breeds (1–4) (Figure S1) and we highlight examples of those found in the within-breed 99.9^th^ percentile that overlapped QTLs and contained biologically interesting genes (Table S1). The Duroc breed exhibited several signatures of diversifying selection on two chromosomes. On SSC14 a highly differentiated region (123.08–123.41 Mb) overlapped with QTLs for fatty acid composition in Duroc [26], [27] and includes a gene involved in fatty acid biosynthesis, *ELOVL3* (123.08–123.083 Mb) [28]. On SSC15 a highly differentiated genomic region (85.73–86.62 Mb) contained the *MYO3B* (Class III myosin B) gene (85.63–85.93 Mb), which directly overlapped the peak F_ST_-window (85.83 Mb). An extended differentiated genomic region was observed in the Landrace breed on SSC13, with the highest F_ST_–window occurring at 73.06 Mb, close to the *GHRL* gene (73.47–73.48 Mb). In addition, QTLs related to various reproductive traits in pigs have been mapped to SSC13 [29] and overlap with the extended differentiated genomic region.

**Figure 1:**
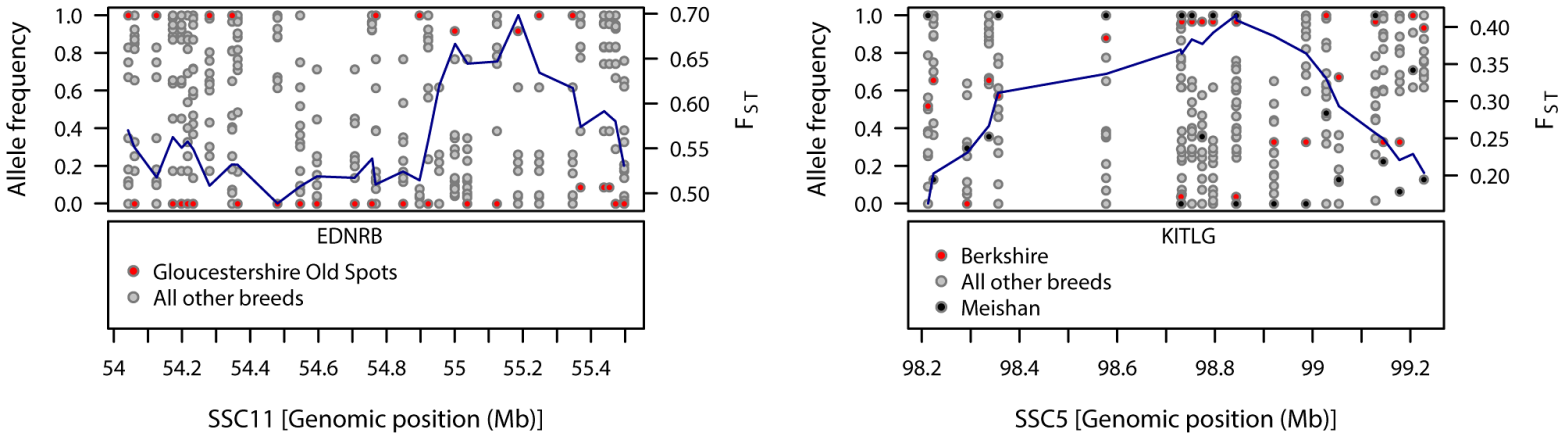
Patterns of genetic variation within regions showing strong signals of diversifying selection in Gloucestershire Old Spots (SSC11) and Berkshire (SSC5). The top-left panel shows the allele frequencies for Gloucestershire Old Spots and the other pig breeds, with F_ST_-windows for Gloucestershire Old Spots shown in blue. The bottom-left panel shows the position of the coat colour gene *EDNRB*. The top-right panel shows the allele frequencies for Berkshire, Meishan and the other pig breeds, with F_ST_-windows for Berkshire shown in blue. The bottom-right panel shows the position of the coat colour gene *KITLG*.

Large, breed-specific signatures of diversifying selection were not limited to the commercial breeds, but also were observed in the traditional breeds (Table S1). Gloucestershire Old Spots displayed a signal of diversifying selection on SSC11, close to *EDNRB* (54.69–54.72 Mb), a gene implicated in coat colour pattern in mammals [30]. Near the peak F_ST_–window (55.20 Mb) many SNPs in this region were fixed in this breed whereas alleles were segregating in all other pig breeds (Figure 1). A weaker signal in the region of this gene (seen in the 99^th^ but not 99.9^th^ percentiles) appeared in Mangalica and British Saddleback breeds (Figure S1). Another breed-specific signature of selection was observed on SSC5 at a different coat colour locus in the Berkshire. *KITLG* (KIT ligand, 98.74–98.78 Mb) was just upstream from a 99.9^th^ percentile F_ST_–window (98.84 Mb) on SSC5 and *KITLG* fell within the 99^th^ percentile differentiation region. Many SNPs in the region of this gene were almost fixed for the same allele in Berkshire and the Asian breed, Meishan, whilst alleles were segregating in the other European pig breeds (Figure 1).

### Phenotypic traits analysis

#### Ear

**Figure 2:**
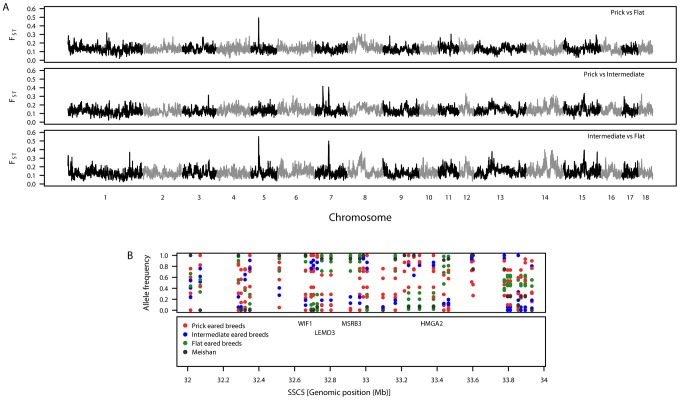
Patterns of genetic variation associated with pig ear phenotypes. A. Genomic distribution of signatures of diversifying selection as measured by genetic differentiation. The top panel shows prick-eared breeds against flat-eared breeds. The second panel shows prick-eared breeds against intermediate-eared breeds. The third panel shows intermediate-eared breeds against flat-eared breeds. B. Variation in breed allele frequencies of SNPs at the candidate region for ear morphology on SSC5. The top panel shows the allele frequencies for each of the European breeds (colour coded by the ear morphology class to which they belong) and Meishan. The second panel shows the positions of biologically interesting genes in that region.

To identify genomic regions associated with ear morphology, we divided the breeds into three classes: prick (upright), intermediate (partly flopped down) and flat (completely flopped down) breeds (Figure S2 and Table S2). Comparisons between these classes revealed three highly differentiated regions on SSC5 and SSC7 (Tables S3, S4, S5; Figure 2A). When prick-eared breeds were contrasted with flat-eared breeds, there was a highly differentiated region on SSC5 (31.74–33.78 Mb) that overlapped with the region identified across eight breeds (see section F_ST_–multiple breeds and Table 2). When prick-eared breeds were contrasted with intermediate-eared breeds, no signal was observed on SSC5 but two signals were observed on SSC7 (31.86–34.19, 55.43–58.19 Mb). When intermediate-eared breeds were contrasted with flat-eared breeds, the same signal was observed on SSC5 (32.28–33.80 Mb) and one of the two signals on SSC7 was present (55.41–58.20 Mb). The peak F_ST_-window on SSC5 overlapped the *LEMD3* gene. The peak F_ST_-window on the second signal on SSC7 occurred at 56.75 Mb, at the *ADAMTSL3* locus (56.50–56.85 Mb). Variation in allele frequencies was observed in the differentiated region on SSC5 overlapping the *LEMD3* gene: alleles were near fixation in the flat-eared breeds, alleles were near fixation for the alternate allele or of intermediate allele frequency in the prick-eared breeds and alleles were segregating in intermediate-eared breeds (Figure 2B).

#### Coat colour

When red coat breeds were compared with non-red coat breeds, the observed outlier regions (Table S6) corresponded with the strong signals of diversifying selection on SSC14 and SSC15 detected in the Duroc from the individual breed comparison (see Figure S1 and Table S1). When black and partially black coat breeds (Large Black, Berkshire, Hampshire, British Saddleback) were compared against red coat breeds, outlier regions were found on 14 chromosomes (Table S7). No signals of selection were detected in the region of *MC1R* (SSC6: 0.26 Mb) for either of these coat colour comparisons.

The next comparison was coat colour phenotypes known to be associated with allelic variation at the *KIT* gene (SSC8: 43.55–43.59 Mb). When belted breeds were compared with non-belted breeds, a differentiated region was observed on SSC8 (41.18–43.08 Mb) near the *KIT* gene (Table S8). However, when non-belted breeds were compared with each other and when belted breeds were compared with each other, a signal of selection was again detected in the region of *KIT*. Although at the location of the *KIT* gene, F_ST_-SNP estimates were higher in the belted vs non-belted breeds comparison than the within-belted breed comparison. When white-coated were compared against non-white-coated breeds a marked differentiation was again observed on SSC8 (43.46–43.73 Mb) in the region of the *KIT* gene, but this was also seen when white-coated breeds were compared against each other and when non-white-coated breeds were compared against each other (Table S9).

#### Teat number

Breeds that had a minimum breed standard of 14 teats were contrasted against breeds where 12 teats was the minimum breed standard. As a form of ‘control’, breeds with 14 teats were compared against one another and breeds with 12 teats were compared against one another. Outlying genomic regions from the 14 teat vs 12 teat comparison that did not overlap with those obtained from the ‘control’ analyses were found on 11 chromosomes (Figure S3 and Table S10). Only one region of several tightly clustered signals on SSC12 (26.83–26.96 Mb; 27.49–32.28 Mb) included genes that could be considered candidates for teat number.

### European breeds versus wild boar

**Figure 3:**
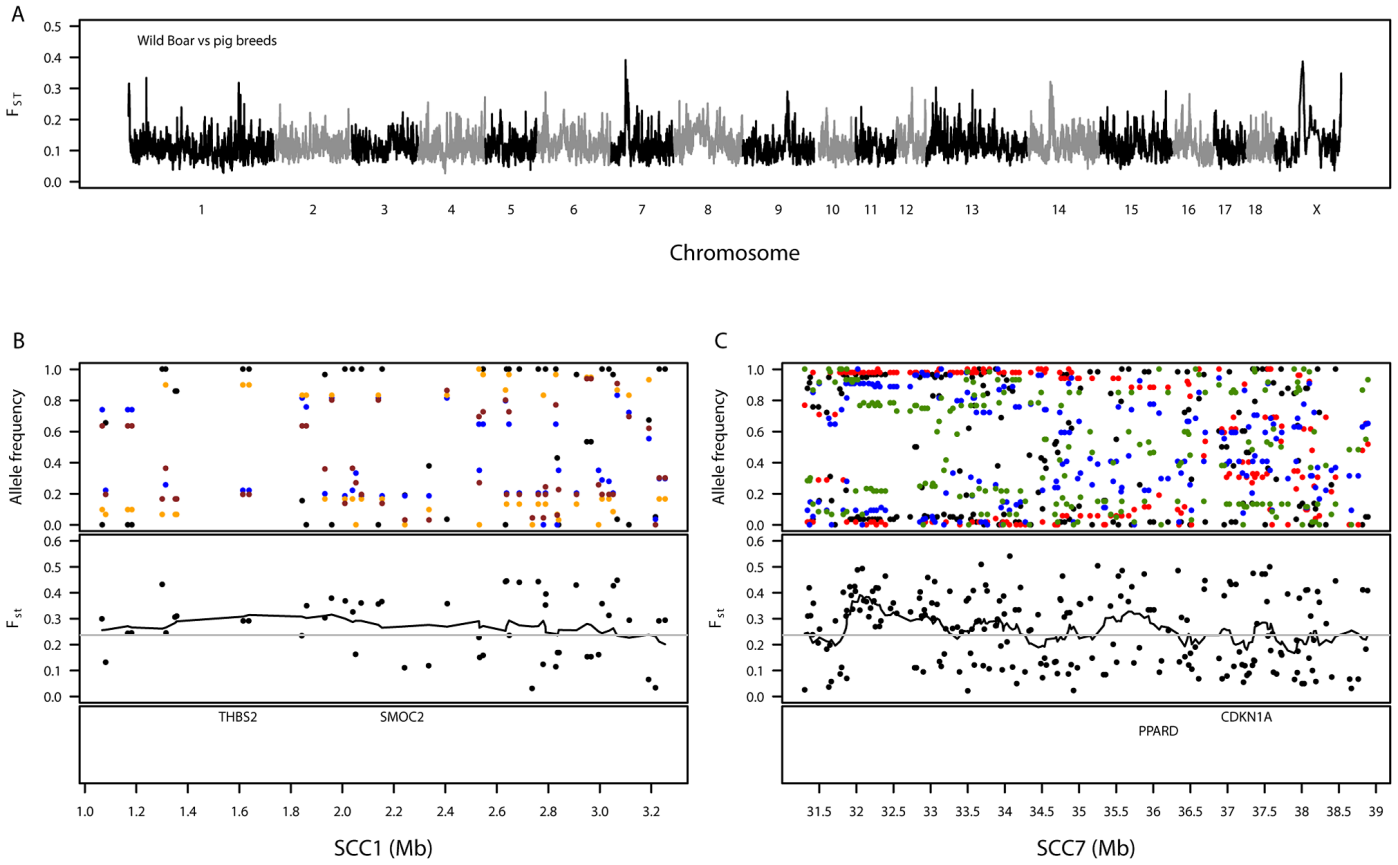
Summary of genetic variation between wild boar and the European pig breeds. A. Genomic distribution of signatures of diversifying selection in pig breeds when contrasted against wild boar. The dashed grey line denotes the 99^th^ percentile. B. Variation in allele frequencies of SNPs compared between wild boar and certain pig breeds on a 2-Mb region on SSC1. The top panel shows the allele frequencies for wild boar (black) versus the Landrace (blue), Welsh (orange) and Tamworth (brown). C. Variation in allele frequencies of SNPs compared between wild boar and certain pig breeds on an 8-Mb region on SSC7. The top panel shows the allele frequencies for wild boar (black) versus the Duroc (red), Landrace (blue) and Large Black (green). For both SSC1 (B) and SSC7 (C), the second panels show the level of genetic differentiation estimated between pig breeds and wild boar and the bottom panels show the positions of biologically interesting genes in these regions.

Levels of genetic differentiation were examined between the European pig breeds and wild boar (Table 1). None of the SNPs were found to be fixed for alternative alleles in the pig breeds and wild boar. The genome-wide distribution of F_ST_ for domestic pig breeds compared with wild boar is shown in Figure 3A. F_ST_–windows falling into the 99^th^ percentile were viewed as candidates of signatures of selection (Table S11) and contained some biologically interesting genes, as described below.

A genomic region on SSC1 showed high levels of differentiation (1.07–3.19 Mb, Table S11), homologous to a region of the canine genome associated with brachycephaly (broad and short skull shape) in dog breeds [31], [32]. This region contains, amongst seventeen characterised and uncharacterised genes, *THBS2* (1.59–1.62 Mb) and *SMOC2* (2.23–2.24 Mb), which were suggested as candidates for brachycephaly in the above-mentioned papers (Figure 3B). Pairwise F_ST_–SNPs between wild boar and each breed in this region (48 SNPs) revealed maximum breed average F_ST_ values for Tamworth (0.42), Welsh (0.43) and Landrace (0.45), none of which have extremely brachycephalic skulls. A highly differentiated genomic region was also observed on SSC7 (31.30–38.89 Mb, Table S11). This region is close to the pig major histocompatibility complex: class I (∼24–26 Mb), class II (∼29 Mb) and class III (∼27 Mb). Within the differentiated region there are several genes of biological interest, including *PPARD* (36.14–36.22 Mb) (Figure 3C). Pairwise F_ST_–SNPs (207) between wild boar and each breed in this region revealed highest breed average F_ST_–SNPs in two commercial breeds, Duroc (0.50) and Landrace (0.37), and one traditional breed, Large Black (0.38); the minimum value of breed average F_ST_ was in Tamworth (0.09). Another interesting differentiation region observed between the domestic pigs and wild boar was on SSCX (Table S11). Amongst other genes, this region contained *AR* (60.31–60.50 Mb), the androgen receptor, previously suggested as a candidate gene for backfat thickness in pigs due to its proximity to mapped QTLs [33]. Other regions showing substantial differentiation between wild boar and pig breeds were found on SSC12, SSC13 and SSC14 but no clear candidate genes could be identified.

### Signals of introgression from Asian pigs into European breeds

Consistent with previous studies [34], [35], genome-wide clustering results indicated substantial Asian ancestry for the European breeds. The clustering results indicated that the inferred ancestry of all Meishan individuals (a breed of Chinese origin, Table 1) to the first (“Asian”) cluster was high (92.3–93.9%). In contrast, the inferred ancestry of the European individuals to the second (“European”) cluster was lower (breed averages ranged from 69.6% for Large White up to 87.3% for Mangalica). With levels of ancestry varying across the genome, regions with particularly strong signals of Asian introgression into European breeds were identified according to two criteria: (1) high introgression probabilities (99^th^ percentile) calculated by STRUCTURE software and (2) low differentiation based on F_ST_ (below the 1^st^ percentile of individual European breeds versus Meishan) (Table S12). Two candidates of introgression overlapped with signals of selection associated with ear morphology. A genomic region on SSC5 (32–35 Mb), overlapping the region of differentiation detected when prick-eared breeds were contrasted with flat-eared breeds, was found in Gloucestershire Old Spots, Large Black and Mangalica (a signal of introgression in British Saddleback, the other flat-eared breed, was observed in this region only in the F_ST_ analysis). A genomic region on SSC7 (33–38 Mb), overlapping with one of the regions of differentiation detected when prick-eared breeds were contrasted with intermediate-eared breeds, was found in British Saddleback, Duroc, Landrace and Welsh. Another signal of introgression was detected on SSC11 (54–55 Mb) in Gloucestershire Old Spots, which overlapped with the differentiated region found in this breed and may be associated with coat pattern. The chromosome with the greatest number of regions showing evidence of Asian introgression was SSC14, where several regions overlapped across multiple breeds (81–85 Mb, eight breeds; 93–94 Mb, four breeds; 96–98 Mb, three breeds; 103–107 Mb, three breeds).

### Sequencing of candidate regions

Based on the differentiation results, three genomic regions were further investigated using genome sequence data for 76 individuals from European and Asian breeds (Table S13).

#### SSC5:31.0–34.0 Mb

We identified 183 variants that were shared by the individuals from flat-eared breeds (British Saddleback, Gloucestershire Old Spots, Large Black and Mangalica) and differed from the individuals from prick-eared breeds (Berkshire, Hampshire, Large White, Middle White, Pietrain and Tamworth). All of these were either intergenic or intronic, with one located 504 bp upstream from a predicted precursor (ENSSSCG00000024846) of microRNA (miRNA) mir-584. However, no EST or RNA-seq evidence could be found in either ENSEMBL or NCBI gene expression data to suggest whether this SNP is located within the primary transcript of mir-584.

#### SSC5:98.0–99.0 Mb

Although the latest ENSEMBL annotation (release 69) predicted two genes in this 1 Mb region, a closer inspection showed that both are parts of the KIT-ligand gene (*KITLG*) but in opposite orientation, indicating probable errors or mis-assemblies here. We therefore blasted the porcine *KITLG* reference mRNA (NM_214269) [36] and an extended 5′-UTR (AB293552, [37]) sequences against the genome to first identify all the exons and the two flanking UTRs, before searching for variants within them.

A single SNP (C1089T), located on the 3′-UTR, was found in both Berkshire individuals but not in any other European breeds. In addition, the two Berkshires were found to harbour 11 other variants that were also present in one or more European breeds. Of these, two were non-synonymous (G548A, A919G) and the remaining nine were on the 5′- or 3′- UTR. The two non-synonymous SNPs resulted in R124K and T248A changes, respectively. The G548A variant was also found in three Pietrains (one a heterozygote) and one Tamworth individual. The A919G variant was also found in individuals of the following breeds: British Saddleback, Gloucestershire Old Spots, Large White, Mangalica, Middle White, Pietrain and Tamworth (two of these, a Pietrain and a Tamworth, also shared the G548 variant). We also examined the sequences of 24 individuals from eight Asian pig breeds and found that all three Jiangquhai individuals carried the C1089T found in the Berkshires, but none of the other Asian individuals carried this variant. The two non-synonymous variants were more common in the Asian than the European breeds: 16/24 Asian individuals carried both of them, compared to 3/50 of the European individuals (excluding the Berkshires).

#### SSC11 53.5–55.5 Mb

The analysed region encompasses 15 annotated genes (11 protein coding plus 4 non-coding RNA). We identified 474 variants in this region that were shared by the two Gloucestershire Old Spots individuals but differed from all other individuals in the European breeds. Of these, one was on the 3′ UTR of an uncharacterised protein-coding gene, three were synonymous variants (in the following genes: *CLN5*, *MYCBP2* and *KCTD12*), and two variants resulted in non-synonymous changes (Table 3), both of which were found in the first exon of the endothelin receptor B (*EDNRB*) gene.

**Table 3:** Description of non-synonymous exonic variants in the 53.5–55.5 Mb region of SSC11.

Location	Nucleotide change	Coding strand	Gene Identifier	Gene name	Transcript	Amino acid change	Region of protein
SSC11:54717799	T49C	-	ENSSSCG00000009477	EDNRB	ENSSSCT00000010390	F17L	signal peptide
SSC11:54717645	C203T	-	ENSSSCG00000009477	EDNRB	ENSSSCT00000010390	S68F	N-terminal extracellular domain

**Figure 4:**
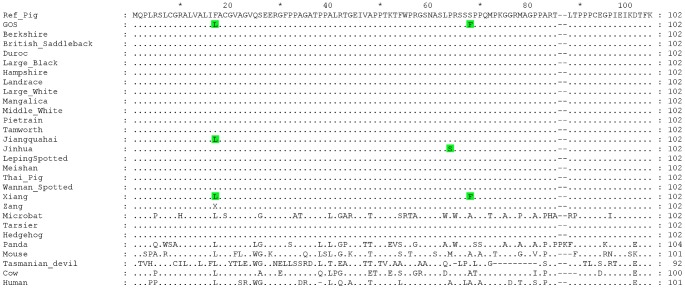
Multiple sequence alignment for the signal peptide and N-terminal extracellular domain of the EDNRB protein. Dots indicate identities to the porcine reference sequence. Accession numbers of sequences used in the alignment: Reference Pig: ENSSSCP00000010120, Microbat: ENSMLUP00000005042, Tarsier: ENSTSYP00000001754, Hedgehog: ENSEEUP00000005222, Panda: ENSAMEP00000005967, Mouse: ENSMUSP00000022718, Tasmanian Devil: ENSSHAP00000009143, Cow: ENSBTAP00000006979, Human: ENSP00000366416.

At residue 17 of EDNRB's signal peptide, the Gloucestershire Old Spots had a leucine (F17L), while the other individuals from European breeds carried a phenylalanine (Table 3, Figure 4). We also examined the *EDNRB* sequences for the Asian breeds and found three individuals (two Xiang, one Jiangquhai) that also carried the leucine, while the rest carried the phenylalanine. The Gloucestershire Old Spots leucine residue, however, was the most common among other mammalian reference genomes (e.g. mouse, cow, hedgehog and human) (Figure 4).

Within the N-terminal extracellular domain of EDNRB, the two Gloucestershire Old Spots individuals carried a phenylalanine at residue 68 (S68F), while a serine was found in the other individuals from European breeds (Table 3, Figure 4). One individual from the Asian Xiang breed also carried phenylalanine, while the other Asian individuals carried serine. There was substantial variability at this site in other mammalian reference proteins but none were found to carry phenylalanine.

Out of the 6928 variants in this region shared by the two Gloucestershire Old Spots, 897 were shared with all individuals from Asian breeds while only 29 were shared with all individuals from the other European breeds.

## Discussion

Over the past 300 years, intense artificial selection in European pig breeds for production traits has led to the development of a number of pig breeds with well-defined, specialised phenotypic traits. In this study a number of regions showing between-breed signatures of selection have been identified. Various genes found within these regions can be considered as candidates under selection based on function or previous association with traits that are known to be favoured in pig breeds.

### Breed standard traits (ear and coat colour)

Signatures of diversifying selection were found for traits related to morphological variation described by breeding criteria. Ear morphology is one trait that plays a major role in pig breed standards with strict conditions over ear form. By grouping breeds based on this phenotypic trait, the genome-wide scan suggested that the genetic basis of ear variation in pigs involves at least three genomic regions, located on SSC5 and SSC7. The region on SSC5 was associated with the difference between prick or intermediate ears and large, flat ears and the signals on SSC7 were associated primarily with the differences between prick- and intermediate-eared breeds. Our results from an introgression analysis also suggest that the SSC5 region of flat-eared breeds derives from Asian pigs.

The signatures of selection associated with ear morphology concurred with an earlier QTL study of the trait in pigs [38]. The SSC7 QTL of Wei et al [38] overlaps directly with the first differentiated region (31.82–34.19 Mb) on that chromosome. The suggestion that *PPARD* located on SSC7 plays a role in ear variation in pig breeds could not be supported as it was not positioned near either of the two signals of selection identified on this chromosome. However, as *PPARD* is involved in many biological processes and is located next to major QTLs for fat deposition and growth, its role in ear morphology warrants further investigation [39]. The QTL peak on SSC5 reported by Wei et al [38] is located approximately 10 Mb upstream of the peak F_ST_ signal but the confidence interval for the QTL location could overlap this position. Genome-wide association studies (GWAS) on ear morphology in dog breeds identified a region underlying this trait that was syntenic to the region on SSC5 in this study [19], [24]. Both these studies suggest *MSRB3* and *HGMA2* as candidate genes due to the proximity of the associated SNP. However, in the pig breeds the peak signal was located closer to *LEMD3*, which is involved in bone morphogenetic protein (BMP) signalling. Recently, a fine mapping study in pigs has suggested *HMGA2* as a candidate locus for this QTL [40]. Mutations in the human version of this gene are associated with disorders involving increased bone density, suggesting a possible role in bone development [41]. However, analysis of coding sequences of these genes in this region of SSC5 for prick- and flat-eared pig breeds did not reveal any shared non-synonymous differences between the two groups, suggesting that changes in regulatory elements or miRNA genes may be responsible. Expression studies are required to test this hypothesis.

Like ear morphology, variation in coat colour patterns occurred post-domestication and signals of selection related to the traits indicate strong historic selection for the different phenotypes. Molecular studies have already identified the major coat colour loci in pigs, *KIT* and *MC1R*, for which allelic variation is associated with many of the coat colour variants (see references in [9], [17]). However, in this study signals of selection were not observed at or near *MC1R* (SSC6) for individual breeds that have an allele associated with a particular coat colour or when breeds were grouped by coat colour traits. The other locus, *KIT* (SSC8), is found ∼1 Mb downstream from a differentiated region shared by three breeds (British Saddleback, Hampshire, Pietrain). Several possible explanations could account for weak and absent signals of diversifying selection at *KIT* and *MC1R*, respectively. The differentiated region on SSC8 was quite extensive in genomic size and *KIT* may have been one of several targets of selection in that region, thus dampening any *KIT*-specific signals. Furthermore, allelic variation at both *KIT* and *MC1R* is associated with a large variety of coat colours and patterns for many breeds. With the breed set analysed in this study, there is no simple dichotomous division of the breeds based on coat type for these two genes, which could have weakened the power of this approach. Lastly, the inter-SNP distances in the *MC1R* region of SSC6 were particularly high (the distance between the flanking markers was in the 99^th^ percentile of the genome-wide distribution of inter-SNP distances). Thus it appears that the *MC1R* region was not well covered by the PorcineSNP60 chip, which may explain why no signals of diversifying selection were detected there.

In contrast to the weak or absent signals of selection at the two major coat colour loci, *KIT* and *MC1R*, strong breed-specific signals of diversifying selection were observed near other coat colour loci. Two non-synonymous mutations were found in the endothelin receptor B (*EDNRB*) gene, in a region exhibiting substantial differentiation unique to Gloucestershire Old Spots. *EDNRB* encodes a G protein-coupled receptor that binds to the different isoforms of endothelins. The EDNRB-endothelin interaction plays a role in a range of critical physiological processes including the formation of enteric nerves and melanocytes (pigment-producing cells), both of which are neural crest derivatives [42], [43].

Mutations in *EDNRB*, leading to a reduced expression of the gene and partial or complete loss-of-function, have been shown to be associated with changes in pigmentation due to its role in melanocyte development [43], [44]. The piebald phenotype in mouse, characterised by white coat spotting [43], results from the insertion of a large retrotransposon in the first intron of *EDNRB* [45]. Several different mutations in humans are associated with a loss of pigmentation in the hair, skin and iris (Hirschsprung's disease/Waardenburg syndrome) [43] while a missense mutation gives rise to the Lethal White Foal Syndrome [46], where homozygous foals are completely white (and die early due to intestinal blockage) while heterozygous animals usually have distinctive white patches.

The mechanism(s) by which point mutations in *EDNRB* could be associated with (partial) loss of function is not yet known. The amino acid changes at residues 64 (Jinhua) and 68 (Gloucestershire Old Spots and Xiang) are both located in the N-terminal extracellular domain of the protein. One of the non-synonymous *EDNRB* mutations associated with Hirschsprung's disease is located in the same domain, at residue 57. This domain has been suggested to be important for stable ligand binding [47]–[49]. Furthermore, human EDNRB is believed to be cleaved by a metalloprotease at R64|S65 (R65|S66 in pig) and a truncated EDNRB (missing the first 64 residues) was found to be functional but had significantly reduced cell surface expression [50]. Using a program that predicts cleavage sites by membrane-type metalloproteases (SitePrediction, [51]), the reference pig EDNRB with S68 was, like its human homologue, found more likely to be cleaved at the R65|S66 site than the Gloucestershire Old Spots protein with F68 (unpublished results). The SNPs that alter residues 64 and/or 68 may result in an incomplete or uncleaved EDNRB and hence altered expression on the cell surface.

Black spotting in the Gloucestershire Old Spots has been previously associated with the *E^P^* allele at the melanocortin receptor 1 (*MC1R* locus): a 2-bp insertion in *MC1R* causes a frameshift mutation which results in a premature stop codon further downstream [15]. That study also demonstrated irregular somatic reversion to the black form of *MC1R* in two spotted breeds, Pietrain and Linderod, such that some regions of the body (black spots) expressed the form of the protein that enables black pigment production, whereas other (white) regions mainly expressed the mutated (non-functional) form of the protein. However, as breeds with various spotted and non-spotted patterns carry the 2-bp insertion, it is likely that additional loci also influence coat pattern and colour in these breeds. A recent paper demonstrated the complex interactions between melanocortin and endothelin signalling in determining coat patterns in cats [52] and similar interactions may also influence coat pattern diversity in pigs. We propose that the variant *MC1R*, resulting from the 2-bp insertion (and somatic reversion), may interact with partial loss of function in *EDNRB* such that only part of the body is populated by melanocytes which have the potential to revert and become pigmented. This in turn could give the Gloucestershire Old Spots its characteristic spotting pattern of relatively few and small spots compared to those observed, for example, in Pietrain. Functional analyses are required to characterize the effects of the Gloucestershire Old Spots variants on *EDNRB* function and on pigmentation patterns.

Although the variants at *EDNRB* were unique to the Gloucestershire Old Spots in the analysis of European breeds, they were shared by the Asian breed Xiang. We do not have phenotypic information for the Xiang individual who shares the Gloucestershire Old Spots variants but one of the most common Xiang subtypes is two-end black with a white middle body, akin to the familiar piebald mouse (http://www.viarural.com.pe/ganaderia/a-porcinos/exteriorcerdos/paises/china.htm). The Jinhua breed, which carries a proline to serine change at nearby residue 64 (Figure 4; [53]), has a similar phenotype. The difference in the phenotypes between the Asian breeds and Gloucestershire Old Spots is likely to be related to their different *MC1R* genotypes. The Asian breeds with *EDNRB* mutations do not carry the *MC1R* insertion (unpublished results), consistent with previous studies that show a low frequency or absence of this allele in Asian pigs [54], [55]. The two Gloucestershire Old Spots individuals are substantially more similar to the Asian breeds than the European ones in the *EDNRB* region. This finding, the shared *EDNRB* genotypes of Gloucestershire Old Spots and Xiang, and the introgression results described above together suggest an Asian origin for the Gloucestershire Old Spots mutations.

A putatively selected region identified in the Berkshire breed includes the *KITLG* locus and further sequence analysis revealed several non-synonymous variants in this breed. KITLG binds to the KIT receptor and plays a role in the melanocyte production pathway. Variation at the locus has been implicated in different skin pigmentation phenotypes in mice (i.e. *steel* mutant) [44], [56] and humans [57], [58], including hypo- and hyper-pigmentation, and has been investigated previously for its role in pig colouration [59]. The breed standard for Berkshire is a black animal with six white points (on the snout, tip of the tail and tips of each of the legs). The Berkshire was allegedly highly variable in coat colour until introgression of Asian genetic material and selection for breed homogeneity led to its contemporary coat pattern [5], [6]. Our tests using PorcineSNP60 data did not detect evidence of Asian introgression for Berkshire in the *KITLG* region (as assessed using comparisons with Meishan), although Berkshire shared the C1089T variant with Jiangquhai, another Asian breed, but not with any other European or Asian individuals. Furthermore, the two non-synonymous variants found in Berkshire were more common in the Asian than the European breeds. Similarly, Okumura and colleagues [37], [60] found evidence for an Asian origin of *KITLG* in Berkshire, as the breed shared haplotypes similar to Asian breeds at the locus whilst differing from other European breeds.

We identified the same two non-synonymous variants (A919G, G458A) in Berkshire and several Asian breeds as Okumura and colleagues [37], [60]. However, these variants cannot on their own explain the Berkshire phenotype because they were also found in three European individuals, including a Pietrain and a Tamworth (both homozygous), the latter breed which is red. Alternatively, the Berkshire phenotype might be attributed to differential regulation of *KITLG*, in conjunction with variation at other pigmentation genes (e.g. *MC1R*—Berkshire also carries the black spotting allele discussed above—and *KIT*). This could be related to the C1089T 3′-UTR variant that was only seen in Berkshire and Jiangquhai (also a black breed) or another regulatory element. *Cis*-regulatory differences in *KITLG* expression have been associated with pigmentation differences in stickleback fish [61] and a SNP located 350 Kb upstream of the *KITLG* gene was found to be associated with human hair colour, suggesting a possible regulatory role [62]. However, we were unable to search for variants in either proximal or distant enhancer/repressor elements due to errors in this region of the current pig genome assembly.

### Pig production traits

Signatures of diversifying selection were found that may be associated with important pig production traits. Teat number is an important reproductive trait because with increased litter size, which is often selected for in pig breeds, a sufficient number of teats are required to support the litter [13]. Although the F_ST_ teat-trait analysis results had some ambiguity, the signal on SSC12 seen in the 14 vs 12 teats comparison but not the ‘control’ comparison (breeds with 14 teats compared with one another and breeds with 12 teats compared with one another) overlapped with documented QTL. Both Hirooka et al [63] and Rodriguez et al [64] reported a significant QTL for teat number on this chromosome, with the latter study suggesting that the most likely position of the QTL was between markers SW874 (23.67 Mb) and SW1956 (40.77 Mb), which overlapped with the region of high differentiation observed in the current study. The *NME1* gene, which is found in this region (27.46–27.50 Mb), plays a role in mammary gland development. *NME1*-deficient mice, although they reproduce normally, have delayed mammary gland development [65] and incomplete maturation of the lactiferous duct in the nipple [66].

Amongst the production characteristics that commercial pig breeds share, they also possess breed-specific characteristics. Duroc pigs are known for their high intramuscular fat content (IMF) in comparison to other commercial pig breeds [67] and for their higher concentrations of saturated and mono-unsaturated fatty acids (and lower concentrations of poly-unsaturated fatty acids) [68], characteristics that play key roles in meat quality. Uemoto et al [27] found a significant QTL for fatty acid composition in Duroc on SSC14 that has not been reported for other breeds. This QTL region overlaps with an extreme differentiation region observed only in the Duroc breed and contains *ELOVL3*, a gene involved in the synthesis of fatty acids; in mice a lack of *ELOVL3* resulted in decreased levels of certain fatty acids due to an inability to convert saturated fatty acyl-CoAs into very long chain fatty acids [28]. In addition, *SCD* (stearoyl-CoA desaturase), a gene located close to the peak differentiation region, encodes a key enzyme in the synthesis of fatty acids and has thus been proposed as a candidate gene for the fatty acid composition QTL [27].

Landrace also exhibited high levels of differentiation, in this case in an extended region of SSC13. The peak differentiation values were found close to the grehlin (*GHRL*) gene, which is a candidate for associations with appetite and feeding behaviour. The regulation of voluntary food intake is controlled by a biological cascade of chemical signals that controls appetite and satiation, where various hormones are involved in the starting and/or termination of an eating episode. Grehlin has been specifically proposed in prompting hunger feelings and therefore initiating eating [69]. Its involvement in regulating feeding behaviour in pigs has only recently been considered [70].

### Genetic signatures underlying domestication

By comparing pig breeds with their ancestral species, the wild boar, we sought to identify genomic regions and genes that could be involved in the domestication process. The largest differentiated genomic region between the domestic pig breeds and wild boar was observed on SSC7. Numerous QTLs have previously been mapped to this chromosome for traits such as growth, carcass length, skeletal morphology and backfat depth using several types of crosses [11], [12]. Several genes located in the region of differentiation have been investigated for possible physiological roles: *PPARD* and *CDKN1A* have been considered candidates for fat deposition [71] and, as mentioned above, *PPARD* has also been considered a candidate gene for ear structure variation [39]. In addition, the genomic signal of selection is close to the MHC region, a complex that is crucial in vertebrate immunity, making it a potential source of evolutionary change on the chromosome. The large differentiated region on SSC7 may reflect strong diversifying selection in domestic pig breeds as this chromosome appears to influence many pig production traits.

Domestic pig breeds are also different from wild boar in skeletal morphology. Substantial changes have occurred in the body and cranial dimensions following domestication [72]. In the comparison of pig breeds with wild boar, a region of genetic differentiation identified on SSC1 is syntenic to a region associated with cranial dimensions in dog breeds [32]. The cranial trait under investigation in the dog studies, brachycephaly, is characterised by a strong alteration of the facial bone structure through shortening of the muzzle and shortening and widening of the skull [31]. Pig breeds possess variable skull morphology ranging from a long snout (Tamworth) to shorter wider faces (Berkshire, Gloucestershire Old Spots, Large Black) to very short faces with upturned snouts, similar to brachycephaly in dogs (Middle White) (see Figure S1). However, Middle White, the most brachycephalic-like breed, did not show significant differentiation from wild boar in the SSC1 region. Incidentally, it has been suggested that Middle White acquired its ‘dished’ face from Asian pigs [6]. However, there was no evidence of Asian introgression into the Middle White in the regions orthologous to the dog brachycephaly regions, suggesting that if it did indeed acquire its squashed face from Asian pigs, there has been independent evolution for this trait in dogs and pigs. As various skeletal and cranial changes occurred after domestication of the wild boar [72], the region of high differentiation overlapping the brachycephaly region in dogs could be associated with other bone alterations.

### Evolutionary perspectives on the development of pig breeds

The putative genomic signatures of selection for breed-defining phenotypic traits and levels of breed genetic differentiation reflect the historical development of the pig breeds. The Duroc had the strongest signals of diversifying selection, evidenced by the levels of genomic differentiation, which were observed to be unique to this breed and unlike the other breeds, no signals of diversifying selection were observed on SSC8 for the Duroc, indicating that this breed may have a distinct genetic origin, as previously noted from microsatellite and sequence data [35], [73]. Some of the clearest signals of both diversifying selection and introgression from Asian pigs were associated with highly visible phenotypes such as coat pattern and ear morphology, suggesting that these traits have been under particularly strong selection during the development of European pig breeds. In particular, selection associated with flat ears was detected in breeds that do not appear to share recent ancestry [7], [73], which may reflect convergent evolution through independent selection for that trait. In contrast, although microsatellite markers indicate a common ancestry for Berkshire and Gloucestershire Old Spots [7], [73], shared differentiation signals were not seen, illustrating differing breed development trajectories. Signatures of selection were also observed in regions associated with certain quantitative traits in pig production, but there was a paucity of signals at loci associated with those related to reproduction. The lack of differentiation signals associated with such traits may reflect their control by many genes of small effect, as suggested by Boyko and colleagues [19].

The genomic regions identified in this study using the genetic differentiation approach generally did not overlap with those identified in a scan for extreme homozygosity in European pig breeds: none of the regions identified in five or more breeds overlapped with the regions reported by Rubin and colleagues [25] and only two out of 109 regions identified in individual breeds overlapped (SSC1:172.13 Mb and SSC15:115.17–115.77 Mb). The Rubin study used more dense genomic data so it is possible that the Porcine SNP60 chip did not contain variants close to the regions they identified. However, in our study we have detected what appear to be genuine signals of selection in pig breed development. Another explanation for the lack of overlap between the studies is that, by pooling genomic data across several breeds, Rubin and colleagues [25] identified regions of homozygosity that were shared amongst the breeds, arguably picking out candidates more likely to be involved in the domestication process and early, post-domestication pig development. In contrast, our methodological approach searched for between-breed differences, thus revealing candidates arising from diversifying selection that occurred during breed development.

## Materials and Methods

### Ethics statement

DNA samples were obtained from blood samples collected by veterinarians according to national legislation, from tissue samples from animals obtained from the slaughterhouse or, in the case of wild boar, from animals culled within wildlife management programs.

### DNA samples, SNP genotyping, and data preparation

DNA samples were obtained from blood samples collected by veterinarians according to national legislation, from tissue samples from animals obtained from the slaughterhouse or in the case of wild boar, from animals culled within wildlife management programs. Samples for SNP genotyping were obtained from between 24 and 34 individuals for 14 pig breeds, described in Table 1, and were genotyped using the PorcineSNP60 chip assay [74]. Most breed samples (including the Asian breed, Meishan) were from the PigBioDiv study whereby a maximum of two individuals were sampled from a litter from as many herds as possible, so as to have as few related individuals as possible in the sample set [75]. For the four commercial breeds (Duroc, Landrace, Large White and Pietrain), the data was from individual commercial populations, which were found to be good representatives of the breeds based on clustering analysis of multiple populations (unpublished results). Welsh samples were provided by the Pedigree Welsh Pig Society. Wild boar samples were those used in the original SNP discovery procedure [74]. Genotype data are deposited in the Dryad repository (http://dx.doi.org/10.5061/dryad.c2124).

All analyses were carried out in R ([76], http://www.r-project.org/). A series of quality control measures were applied to the dataset to filter out any possible genotyping anomalies. First, SNP markers that had greater than 10% missing genotypes were discarded. Second, markers that were monomorphic across all the breeds (i.e. MAF<0.01) were also discarded from further analysis. Third, SNP markers were tested for deviations from Hardy-Weinberg equilibrium within each breed using an exact test [77]. At a critical rejection region of 8.33×10^−7^ (0.05/60,000) a total of 66 SNPs did not conform to HWE expectations in one or more breeds and were removed from the analysis. Of these, 46 deviated from HWE due to excess of heterozygote genotypes in one or more breeds. The other 20 SNPs deviated from HWE due to heterozygote deficit in one or more breeds. Fourth, markers that were not mapped to the porcine genome were removed, based on the current pig genome assembly, *Sus scrofa* (SSC) Build 10.2. For the remaining markers, SNPs that were not yet mapped to a specific location on a specific chromosome of the pig genome were also filtered out. Following quality control, 49 260 markers were considered for the majority of analyses (see below for one exception). After QC, average individual genotype coverage was 99.20% across all breeds and average individual genotype coverage in individual breeds ranged from 96.09% in the Mangalica breed to 99.96% in the Hampshire breed.

### Statistical analysis

Pairwise Wright's F_ST_ [78], the classical measure of population genetic differentiation, was used to detect signatures of diversifying selection. We previously showed [79] that pairwise measures of differentiation were better at identifying markers that distinguished breeds than global measures and that Wright's estimate of F_ST_ was highly correlated to that of Weir & Cockerham's [80]. The use of population (breed) differentiation to identify candidate selected regions, as implemented in the current study, was originally suggested by Akey and colleagues [81]. This approach was justified by use of simulations in a follow-up study on dogs [18] and has subsequently been implemented in various empirical studies [22], [24], [82].

The PorcineSNP60 chip assay was designed to include SNPs evenly distributed across the genome, with per-chromosome average inter-SNP distances ranging from 30 to 40 kb (except for SSCX) (based on builds 7 and 8) [74], with a median of 30 kb for the genome-wide distribution. Across the genome, the majority (80%) of inter-SNP distances were less than 70 kb in this study. Recent studies (e.g. Ref. [83]) show high linkage disequilibrium across commercial pig genomes (r^2^∼0.4 between adjacent SNPs on the PorcineSNP60 chip), suggesting that our study is likely to detect most signals. To account for stochasticity in locus-by-locus variation, for all of the F_ST_ analyses a 13-SNP sliding window was implemented on the estimated values, with the mid-SNP determining the genomic location of the window (hereafter designated as F_ST_-window). To allow 13-SNP sliding windows across a whole chromosome, the first window on a chromosome was centred at the 7^th^ SNP position and the last window on a chromosome was centred at the 7^th^ from last SNP position. Candidate selected regions were defined as the 99^th^ percentile of the empirical distributions of F_ST_-windows, except where indicated otherwise.

### Individual pig breeds

A breed average F_ST_ was first calculated. F_ST_ was estimated between pairs of European breeds at each SNP marker using the breed allele frequencies. For each breed this produced 12 breed-pairwise F_ST_ comparisons at each SNP marker. The F_ST_ at each SNP marker for all of these pairwise comparisons were averaged to produce an overall F_ST_ for each SNP marker in each breed (here after designated as F_ST_-SNP).

### Phenotypic traits

The F_ST_ analysis was extended to compare groups with different phenotypic traits. For each trait classes were formed, based on the observed phenotypic variation between breeds (see below), and breeds were placed into one of the classes. For each trait, F_ST_ was estimated between each breed in one class compared against each breed in the next class and averaged across the pairwise comparisons to obtain a F_ST_-SNP estimate. A summary table of the different traits, the phenotypic classes and the class designation of each breed is shown in Table S2.

Ear morphology in European pigs is variable, ranging from upright or prick ears that may be slightly inclined forwards (the ancestral state as seen in wild boar), to a medium sized ear that points forwards and downwards but is not too heavy, to a completely dropped ear that is long, thin and lies relatively flat against the face slightly curbing vision of the animal (see Figure S2). Ear morphology was grouped into the following classes: prick-eared breeds, intermediate-eared breeds and flat-eared breeds.

Coat colour in European pigs is a highly variable phenotypic trait including from black, red, brown and white, with and without spots and belts. The coat colour was grouped into the following classes: red coat breeds compared with non-red coat breeds; saddleback breeds compared with non-saddleback breeds; white coat breeds compared with non-white coat breeds; red coat breeds compared with black coat breeds.

Amongst the breed standard requirements set by the British Pig Association (BPA), the number of teats is one listed criterion. Using this trait, breeds were grouped in the following classes: breeds where the BPA standards required a minimum of 14 displayed teats compared with breeds where the BPA standards required a minimum of 12 displayed teats, Berkshire and Middle White were removed from this trait comparison because there was not a definitive breed standard requirement (breed standards suggested a “minimum of 12 but preferably 14 teats”) and Mangalica was also removed because the breed standard number of teats was unknown.

### Pig breeds versus wild boar

Levels of genetic differentiation between the domestic pig breeds and wild boar were estimated. The SNPs that were monomorphic in the pig breeds were compared with wild boar genotypes to determine if some were segregating in the wild boar. The (mapped) breed-monomorphic SNPs that were segregating in the wild boar were added to the set of polymorphic SNPs described above, giving a total of 49 556 markers. F_ST_ was estimated between wild boar and each pig breed, which produced 13 pairwise comparisons at each SNP marker. The F_ST_ at each SNP marker for each of these pairwise comparisons were averaged to produce an overall F_ST_ for each SNP marker (here after designated as F_ST_-SNP).

### Signals of Asian introgression into European breeds

Two methods were employed to infer signals of Asian introgression in European breeds. First, an F_ST_ analysis, as described above, was used to quantify differentiation between the Asian Meishan breed and each of the 13 European breeds. Regions of particularly low differentiation (below 1^st^ percentile) were interpreted as showing evidence of Asian introgression.

Second, a Bayesian analysis was performed using the site-by-site linkage model in STRUCTURE software [84]. This model was designed to infer the ‘population-of-origin’ assignment of genomic regions and has been used to determine levels of introgression between populations (e.g. Ref. [85]). Each of the 13 European breeds was compared with the Meishan breed, using no *a priori* population information: at a pre-defined number of clusters, K = 2, the linkage model was run five times for 20,000 iterations after a burn-in of 40,000 iterations (which included 20,000 iterations with the admixture model). Due to computer memory limitations, for the analysis 15 individuals per breed (approximately half of the total dataset) were chosen at random and every second marker across each chromosome was removed from the input data set leaving a total of 24 630 markers.

Ancestry proportions across the two clusters (“Asian” and “European”) were estimated for each of the European individuals. Estimates of Asian ancestry for each European animal for each SNP were obtained from the probability of assignment to the Asian cluster and then averaged across the individuals within each breed. As described above, a sliding window average of Asian ancestry values across each chromosome was calculated, with windows composed of 7 SNPs (half the number used for the analyses of the full set of SNPs). The average value for the window was assigned to the position of the central SNP. These values were interpreted as probabilities of introgression from Asian to European breeds.

In order to identify genomic regions with clear signals of Asian introgression, we identified SNP positions (to the closest Mb) that met two criteria: (1) values below the 1^st^ percentile of the Meishan-European breed F_ST_-windows distribution and (2) found in the 99^th^ percentile of STRUCTURE-calculated introgression probabilities for that breed.

### Sequencing strategy

DNA samples for sequencing were obtained as described above for SNP genotyping. Individual samples (52) from 12 of the European breeds analysed above (no Welsh pigs were included) as well as 24 samples from eight Asian breeds (Table S13) were sequenced using the Illumina HiSeq2000 platform, with library preparation and sequence generation per manufacturers protocols. Sequence mapping and variant calling were carried out as described previously [25], [34]. Briefly, Illumina (v. 1.3–1.8) formatted fastq files, with sequence reads of 100 bp were subject to quality trimming prior to sequence alignment. The trimming strategy involved a 3 bp sliding window, running from 5′ to 3′, with sequence data upstream being discarded if the 3 bp window average quality dropped below 13 (i.e. average error probability equal to 0.05). Only sequences of 45 bp or more in length were retained. In addition, sequences with mates <45 bp after trimming were also discarded. During trimming, quality scores were re-coded to follow the Sanger fastq format to standardize downstream processing.

Sequences were aligned against the Sscrofa10.2 reference genome using Mosaik 1.1.0017. Alignment was performed using a hash-size of 15, with a maximum of 10 matches retained, and 7% maximum mismatch score, for all pig populations and outgroup species. Alignment files were then sorted using the MosaikSort function, which entails removing ambiguously mapped reads that are either orphaned or fall outside a computed insert-size distribution. Alignment archives were converted to BAM format using the Mosaiktext function. Manipulations of BAM files, such as merging of alignments archives pertaining the same individual, were conducted using SAMtools v. 1.12a [86].

Variant allele calling was performed per individual using the pileup function in SAMtools, and variations were initially filtered to have minimum quality of 50 for indels, and 20 for SNPs. In addition, all variants showing higher than 3x the average read density, estimated from the number of raw sequence reads, were also discarded to remove false positive variant calling originating from off-site mapping as much as possible. Heterozygous variants and those with minimal SNP/indel qualities were further inspected manually to ensure that they were true variants.

We examined the sequence variation in three genomic regions that showed extreme differentiation in one or more breeds (Table S1) for the individuals from the 12 European breeds: (1) SSC5:31.0–34.0, (2) SSC5:98.0–99.0 and (3) SSC11:53.5–55.5 Mb. Information for the relevant regions was excised from the BAM files using SAMtools v. 1.12a [86]. Alignment files and variants called in these regions for all animals considered in this manuscript are deposited in the Dryad repository (http://dx.doi.org/10.5061/dryad.c2124). For the first region, we identified all variants that were shared by the individuals from flat-eared breeds but differed from all individuals from the prick-eared breeds (Table S2); for the second region, we identified all variants that were shared by the two Berkshire individuals but differed from the other individuals; and for the third region, we identified all variants that were shared by the two Gloucestershire Old Spots individuals but differed from the other individuals. Data for the individuals from Asian breeds was then used to examine specific sequence variants, as described in the Results.

## Supporting Information

Figure S1 Genome-wide distribution of signatures of diversifying selection in the pig breeds measured by genetic differentiation. The F_ST_-window across all chromosomes is shown for each breed. The dashed grey line denotes the 99^th^ percentile for each breed. Breeds are abbreviated as described in Table 1.
(EPS)

Figure S2 Example photos of the ear structure of breeds for the three classes of ear morphology trait.
(DOC)

Figure S3 Genome-wide distribution of signatures of selection for the trait comparison of number of teats. The top panel plots the overlap of the 99^th^ percentile for each of the following comparisons: 14 teats vs 12 teats, 14 teats vs 14 teats and 12 teats vs 12 teats. The second panel shows the 14 teat breeds compared with 12 teat breeds. The third panel shows the 14 teat breeds compared against each other. The final panel shows the 12 teat breeds compared against each other.
(EPS)

Table S1 Summary of genomic regions exhibiting diversifying selection in individual breeds. F_ST_-windows that were found in the 99.9^th^ percentile of values identified in individual breeds were deemed as outliers. Regions separated by more than 5 markers are listed individually.
(XLS)

Table S2 Summary of the phenotypic traits, the classes and breeds assigned to classes used in the F_ST_ trait analysis. Breed: see abbreviations on Table 1; Ear: PR = Prick-eared, INT = Intermediate-eared and FLAT = Flat-eared breeds, see Materials and Methods for description for each ear class; Teat: 12 = a minimum of 12 teats required by BPA breed standards and 14 = a minimum of 14 teats required by BPA breed standards; Red: RED = red-coat breed and N = non-red-coat breed; Belt: BE = belted breed and N = non-belted breed; White: WH = white-coat breed and N = non-white-coat breed.
(DOC)

Table S3 Summary of genomic regions exhibiting diversifying selection in the ear trait analysis: prick vs flat. F_ST_-windows that were found in the 99^th^ percentile of values identified in the trait comparison were deemed as outliers. Regions separated by more than 5 markers are listed individually.
(XLS)

Table S4 Summary of genomic regions exhibiting diversifying selection in the ear trait analysis: prick vs intermediate. F_ST_-windows that were found in the 99^th^ percentile of values identified in the trait comparison were deemed as outliers. Regions separated by more than 5 markers are listed individually.
(XLS)

Table S5 Summary of genomic regions exhibiting diversifying selection in the ear trait analysis: intermediate vs flat. F_ST_-windows that were found in the 99^th^ percentile of values identified in the trait comparison were deemed as outliers. Regions separated by more than 5 markers are listed individually.
(XLS)

Table S6 Summary of genomic regions exhibiting diversifying selection in the coat colour trait analysis: red breeds versus non-red breeds. F_ST_-windows that were found in the 99^th^ percentile of values identified in the trait comparison were deemed as outliers. Regions separated by more than 5 markers are listed individually.
(XLS)

Table S7 Summary of genomic regions exhibiting diversifying selection in the coat colour trait analysis: black breeds versus red breeds. F_ST_-windows that were found in the 99^th^ percentile of values identified in the trait comparison were deemed as outliers. Regions separated by more than 5 markers are listed individually.
(XLS)

Table S8 Summary of genomic regions exhibiting diversifying selection in the coat colour trait analysis: belted breeds versus non-belted breeds. F_ST_-windows that were found in the 99^th^ percentile of values identified in the trait comparison were deemed as outliers. Regions separated by more than 5 markers are listed individually.
(XLS)

Table S9 Summary of genomic regions exhibiting diversifying selection in the coat colour trait analysis: white breeds versus non-white breeds. F_ST_-windows that were found in the 99^th^ percentile of values identified in the trait comparison were deemed as outliers. Regions separated by more than 5 markers are listed individually.
(XLS)

Table S10 Summary of genomic regions exhibiting diversifying selection in the teat trait analysis: 14 teats vs 12 teats. F_ST_-windows that were found in the 99^th^ percentile of values identified in the trait comparison were deemed as outliers. Regions separated by more than 5 markers are listed individually.
(XLS)

Table S11 Summary of genomic regions exhibiting differentiation between wild boar and individual pig breeds. F_ST_-windows that were found in the 99^th^ percentile of values identified in the comparison of wild boar vs individual European breeds were deemed as outliers. Regions separated by more than 5 markers are listed individually.
(XLS)

Table S12 Summary of genomic positions to the closest 1 Mb that had both outlier (99^th^ percentile) Asian introgression probabilities calculated by STRUCTURE and outlier (below 1^st^ percentile) F_ST_-windows identified in comparisons of Meishan vs individual European breeds.
(XLSX)

Table S13 Numbers of individuals for which sequence data was analysed from three target regions.
(DOCX)
