# Supplementary material for: Signatures of Diversifying Selection in European Pig Breeds
Source: PLoS Genet. 2013 Apr 25;9(4):e1003453. doi: 10.1371/journal.pgen.1003453 (PMC3636142; doi:10.1371/journal.pgen.1003453)
Supplement: Figure S2 — Example photos of the ear structure of breeds for the three classes of ear morphology trait. (DOC) [file pgen.1003453.s002.doc]

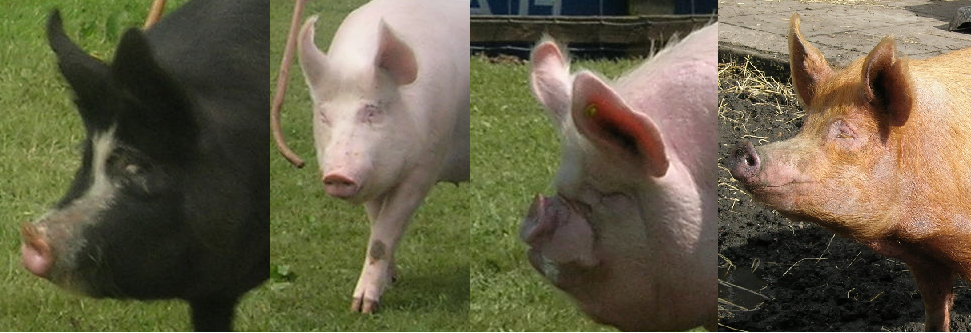


Prick eared breeds: Berkshire, Large White, Middle White and Tamworth from left to right.


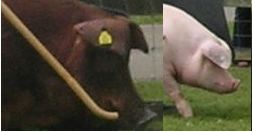


Intermediate eared breeds: Duroc and Landrace from left to right.


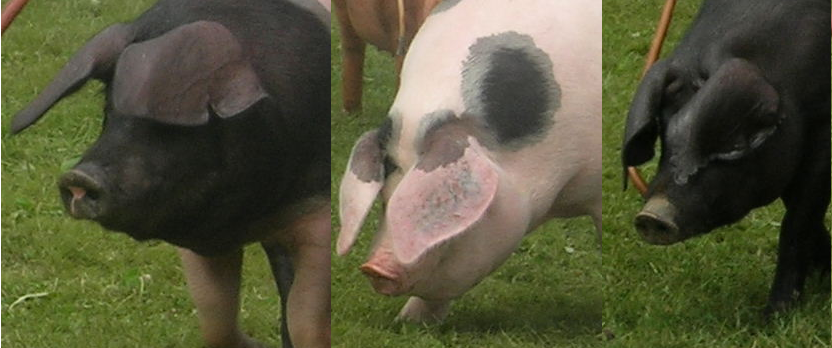


Flat eared breeds: British Saddleback, Gloucestershire Old Spots and Large Black from left to right.

Supplementary Figure S2 Example photos of the ear structure of breeds for the 3 classes of ear morphology trait (digital photos taken by S Wilkinson).
