# Supplementary material for: Signatures of Diversifying Selection in European Pig Breeds
Source: PLoS Genet. 2013 Apr 25;9(4):e1003453. doi: 10.1371/journal.pgen.1003453 (PMC3636142; doi:10.1371/journal.pgen.1003453)
Supplement: Table S2 — Summary of the phenotypic traits, the classes and breeds assigned to classes used in the FST trait analysis. Breed: see abbreviations on Table 1; Ear: PR = Prick-eared, INT = Intermediate-eared and FLAT = Flat-eared breeds, see Materials and Methods for description for each ear class; Teat: 12 = a minimum of 12 teats required by BPA breed standards and 14 = a minimum of 14 teats required by BPA breed standards; Red: RED = red-coat breed and N = non-red-coat breed; Belt: BE = belted breed and N = non-belted breed; White: WH = white-coat breed and N = non-white-coat breed. (DOC) [file pgen.1003453.s005.doc]

| Breed | Ear | Teat | Red | Belt | White |
| --- | --- | --- | --- | --- | --- |
| BK | PR | - | N | N | N |
| BS | FLAT | 12 | N | BE | N |
| DU | INT | 12 | RED | N | N |
| GLOS | FLAT | 14 | N | N | N |
| HA | PR | 12 | N | BE | N |
| LR | INT | 14 | N | N | WH |
| LB | FLAT | 12 | N | N | N |
| LW | PR | 14 | N | N | WH |
| MA | FLAT | - | N | N | N |
| MW | PR | - | N | N | WH |
| PI | PR | 12 | N | N | N |
| TA | PR | 12 | RED | N | N |
| W | INT | 14 | N | N | WH |

Supplementary Table S2 Summary of the phenotypic traits, the classes and breeds assigned to classes used in the F_ST_ trait analysis. Breed: see abbreviations on Table 1; Ear: PR = Prick-eared, INT = Intermediate-eared and FLAT = Flat-eared breeds, see Materials and Methods for description for each ear class; Teat: 12 = a minimum of 12 teats required by BPA breed standards and 14 = a minimum of 14 teats required by BPA breed standards; Red: RED = red-coat breed and N = non-red coat breed; Belt: BE = belted-breed and N = non-belted breed; White: WH = white-coat breed and N = non-white coat breed.
