# Supplementary material for: Signatures of Diversifying Selection in European Pig Breeds
Source: PLoS Genet. 2013 Apr 25;9(4):e1003453. doi: 10.1371/journal.pgen.1003453 (PMC3636142; doi:10.1371/journal.pgen.1003453)
Supplement: Table S13 — Numbers of individuals for which sequence data was analysed from three target regions. (DOCX) [file pgen.1003453.s016.docx]

Table S13. Numbers of individuals sequenced in three target regions (see text for details).

European breeds

Berkshire 2

British Saddleback 1

Duroc 7

Gloucestershire Old Spots 2

Hampshire 4

Landrace 6

Large Black 1

Large White 16

Mangalica 2

Middle White 1

Pietrain 8

Tamworth 2

Asian breeds

Jinhua 2

Jiangquhai 3

Leping Spotted 2

Meishan 10

Thai Pig 2

Wannan Spotted 2

Xiang 2

Zhang 1
